# Compressional-Puffing Pretreatment Enhances Neuroprotective Effects of Fucoidans from the Brown Seaweed *Sargassum hemiphyllum* on 6-Hydroxydopamine-Induced Apoptosis in SH-SY5Y Cells

**DOI:** 10.3390/molecules23010078

**Published:** 2017-12-29

**Authors:** Chun-Yung Huang, Chia-Hung Kuo, Po-Wei Chen

**Affiliations:** Department of Seafood Science, National Kaohsiung Marine University, No. 142, Haijhuan Rd., Nanzih District, Kaohsiung 81157, Taiwan; chkuo@webmail.nkmu.edu.tw (C.-H.K.); a3989889@gmail.com (P.-W.C.)

**Keywords:** 6-hydroxydopamine, antioxidant, apoptosis, fucoidan, human neuroblastoma SH-SY5Y cells, neuroprotection, *Sargassum hemiphyllum*

## Abstract

In this study, a compressional-puffing process (CPP) was used to pretreat *Sargassum hemiphyllum* (SH) and then fucoidan was extracted from SH by hot water. Three fucoidan extracts, namely SH1 (puffing at 0 kg/cm^2^); SH2 (puffing at 1.7 kg/cm^2^); and SH3 (puffing at 10.0 kg/cm^2^) were obtained, and their compositions and biological activities were evaluated. The results indicate that CPP increased the extraction yield, total sugar content, and molar ratios of sulfate/fucose of fucoidan and decreased molecular weight and impurities of fucoidan. The SH1–SH3 extracts exhibited characteristics of fucoidan as demonstrated by the analyses of composition, FTIR spectroscopy, NMR spectroscopy, and molecular weight. All SH1–SH3 extracts showed antioxidant activities. The SH1–SH3 extracts protected SH-SY5Y cells from 6-hydroxydopamine (6-OHDA)-induced apoptosis as illustrated by cell cycle distribution, cytochrome c release, activation of caspase-8, -9, and -3, and DNA fragmentation analyses. Additional experiments revealed that phosphorylation of Akt is involved in the opposing effects of SH1–SH3 on 6-OHDA-induced neurotoxicity. SH3 exhibited a relatively high extraction yield, the lowest levels of impurities, and was the most effective at reversing the 6-OHDA-induced neurotoxicity of SH-SY5Y cells among SH1–SH3, which taken together indicate that it may have potential as a candidate therapeutic agent for the preventive therapy of neurodegenerative diseases.

## 1. Introduction

Populations worldwide are aging and as a result the prevalence of neurodegenerative diseases is increasing. These neuronal disorders are classified into two pathological classes: Movement disorders such as Parkinson’s disease (PD), and cognitive deterioration and dementia, such as Alzheimer’s disease (AD) [1]. PD and AD can be attributed to neuronal damage which results in loss of memory and cognition or movement disorder. The underlying mechanisms are thought to involve increased free radical damage due to oxidative stress [2]. In fact, the brain and nervous systems are susceptible to oxidative damage. A variety of evidence has demonstrated that oxidative stress-induced cell damage leads both to the physiological process of aging and to the progression of AD and PD [1]. Therefore, there is a clear need for naturally derived antioxidants that may have the potential to serve as chemopreventive agents for neurodegenerative diseases.

Brown seaweeds contain large amounts of polysaccharides in their cell walls including fucoidans, cellulose, and alginic acids. Fucoidans are a group of fucose-containing sulfated polysaccharides (FCSPs) comprising mixtures of structurally related polysaccharides with certain variations of monosaccharide residues and containing non-carbohydrate substituents (mainly sulfate and acetyl groups) [3]. Fucoidans can be extracted from brown seaweeds such as *Sargassum* spp. [4]. These fucoidans have been widely documented to exhibit multiple biological functions including antioxidant, antivirus, anti-inflammatory, antitumor, and antithrombotic and anticoagulant effects [4,5]. However, relatively few studies on the neuroprotective effects of fucoidans from *Sargassum* spp. have been reported. Thus, we aimed to find extracts of fucoidan from *Sargassum* spp., and to study their effects on neuroprotective functions.

This study builds upon the work of our previous research [6,7]. Briefly, a brown seaweed *Sargassum hemiphyllum* (SH), after being washed and oven-dried, was compressional-puffed at various pressures and then the crude extracts of fucoidans were extracted by hot water. The extraction yield, composition, structure, antioxidant, and neuroprotective functions of crude extracts of fucoidan were examined. To the best of the authors’ knowledge, no such studies have been reported in the literature relating to the reversal of 6-hydroxydopamine (6-OHDA)-induced apoptosis in SH-SY5Y cells by crude extracts of fucoidan extracted from compressional-puffing-pretreated SH. In addition, we explored the potential of fucoidan from SH to serve as natural chemopreventive agents for preventive therapy of neurodegenerative diseases, especially PD.

## 2. Results and Discussion

### 2.1. Effects of Compressional-Puffing Parameters on the Degree of Moisture Loss of Puffed Algal Samples and Extraction Yields of Fucoidan

The algal sample of SH used in this study was collected from Pingtung, Taiwan, and contained 7.05% ± 0.30% protein, 1.01% ± 0.01% lipid, 26.70% ± 0.16% ash, and 65.24% ± 0.13% carbohydrate (dry basis). The chemical composition data indicate that SH possessed a relatively high amount of carbohydrate (more than 50%), and thus it was considered suitable for extraction of fucoidan. Before extraction of fucoidan, the algal sample was pretreated with a compressional-puffing process (CPP). The CPP has been proven to effectively increase the extraction yields of fucoidan from brown seaweeds [6,7] and to augment the extraction yields of total phenolics and total flavonoids from pine needles [8,9]. Table 1 shows the operational parameters for CPP, which include mechanical compression pressure, number of compression times, puffing temperatures, and reaction time. Afterwards, the powder of SH (weight 2.7 g, H_2_O = 12.9%) was heated and puffed at 140 and 180 °C, which correspond to the pressures inside the chamber, 1.7 and 10.0 kg/cm^2^, respectively (Table 1). CPP essentially involves three stages. In the first stage, when the temperature of the plate reaches the setting temperature, the upper plate comes down to the bottom plate. In the second stage, the upper plate applies mechanical pressure on the bottom plate three times. In the final stage, the upper plate returns to its original position which results in a sudden release of the high pressure steam, completing the process. The degree of moisture loss in the puffed algal sample is shown in Table 1. When the pressure reached 1.7 kg/cm^2^, the moisture loss for SH2 was 16.21% ± 1.17%. When the pressure was increased to 10.0 kg/cm^2^, the moisture loss for SH3 was 29.56% ± 2.21%. Thus, the degree of moisture loss in puffed algal sample was significantly increased by elevating the puffing pressures (*p* < 0.05). We subsequently obtained fucoidans from the compressional-puffed algal samples by 85 °C water extraction, removal of alginate and protein, 50% ethanol precipitation, and lyophilization. Table 1 shows the extraction yields of fucoidan for SH1, SH2, and SH3, and the data were 1.51% ± 0.09%, 1.93% ± 0.28%, and 2.06% ± 0.14% (dry basis), respectively, by setting the puffing pressures at 0, 1.7, and 10.0 kg/cm^2^, respectively, indicating that CPP could raise the extraction yields of fucoidan. These extraction yield data in the present study surpassed our previous findings which indicated that fucoidan extracted from compressional-puffed *S*. *crassifolium* possessed extraction yields ranging from 0.68% to 1.08% (dry basis) [7]. In conclusion, our data showed that CPP pretreatment effectively increased the extraction yields of fucoidan in algal samples.

### 2.2. Compositional and Physicochemical Analyses of Fucoidans for SH1, SH2, and SH3

**Figure 1:**
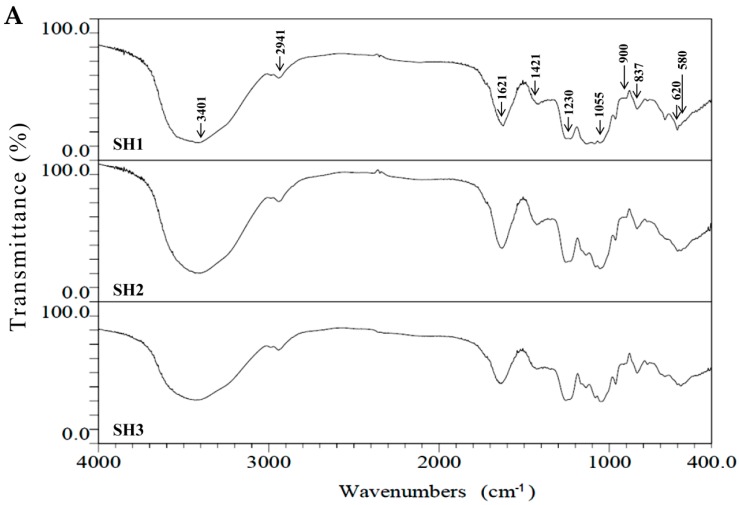

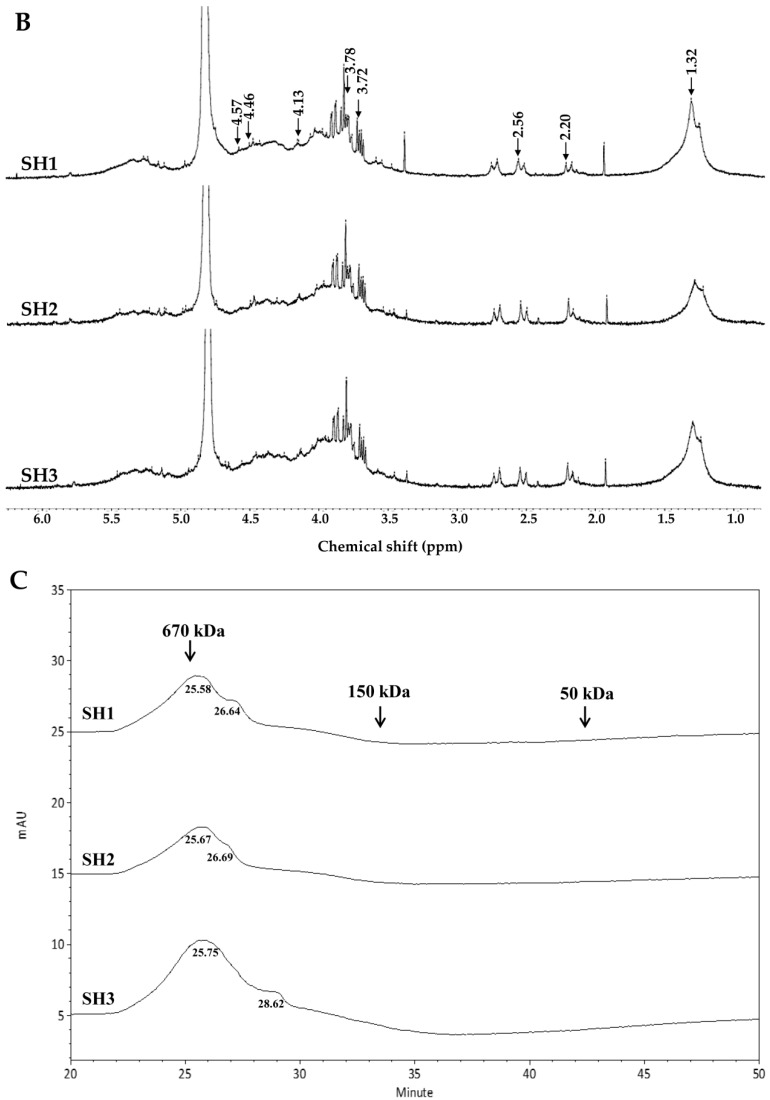
Characteristics of fucoidan extracts obtained from *S. hemiphyllum* under different compressional-puffing pretreatments. (**A**) FTIR spectra for SH1, SH2, and, SH3. Absorption bands at 3401, 2941, 1621, 1421, 1230, 1055, 900, 837, 620, and 580 cm^−1^ are indicated; (**B**) ^1^H-NMR spectra for SH1, SH2, and SH3. Chemical shifts at 4.57, 4.46, 4.13, 3.78, 3.72, 2.56, 2.20, and 1.32 ppm are indicated; (**C**) size exclusion chromatographic profiles for SH1, SH2, and SH3. Dextrans with molecular weights 670, 150, and 50 kDa were utilized as the standards.

The compositional analyses of fucoidan extracts for SH1–SH3 were performed and the results are presented in Table 2. The total sugar contents for SH1, SH2, and SH3 were 53.67% ± 2.59%, 58.83% ± 1.61%, and 68.83% ± 1.91% *(w*/*w*, dry basis), respectively, and were upregulated by raising the puffing pressures. The total sugar contents of SH1–SH3 were comparable to the amount (61%, dry basis) of polysaccharide extracted from brown alga *S. tenerrimum* in a previous study [10]. The uronic acid contents for SH1–SH3 ranged from 5.66% ± 0.08% to 7.04% ± 0.22% *(w*/*w*, dry basis) (Table 2), and did not appear to be related to the puffing pressures. The fucose contents for SH1–SH3 ranged from 22.76% ± 0.38% to 24.53% ± 1.15% *(w*/*w*, dry basis) (Table 2), suggesting that CPP did not obviously affect the fucose content. The sulfate content of fucoidan plays a critical role in the biological functions as previously noted by other investigators [11,12]. We thus measured the sulfate contents for SH1, SH2, and SH3 and the percentages were 18.87% ± 1.65%, 15.58% ± 0.16%, and 24.16% ± 2.80% (*w*/*w*, dry basis), respectively (Table 2), and found that SH3 possessed the highest sulfate content among SH1–SH3. The molar ratio of sulfate/fucose in fucoidan was shown to play a pivotal role in the bioactivities [13]. We therefore calculated the molar ratios of sulfate/fucose in SH1, SH2, and SH3 and the results were as follows: 1.32 ± 0.11, 1.17 ± 0.01, and 1.74 ± 0.20, respectively (Table 2). The trend was similar to that of sulfate content, and SH3 had the highest molar ratio of sulfate/fucose among SH1–SH3. Thus, it was expected that SH3 may have high biological activity, and further investigation was warranted. The co-extracted substances such as alginate, protein, and polyphenols can be regarded as impurities [6]. The alginate was not detected in SH1–SH3. From Table 2, the total amount of protein and polyphenol impurities of fucoidan can be calculated for SH1 (2.28 + 1.25 = 3.53, g/100 g, dry basis), SH2 (2.05 + 1.06 = 3.11, g/100 g, dry basis), and SH3 (1.83 + 0.71 = 2.54, g/100 g, dry basis), and the impurity contents were downregulated by elevated puffing pressures. It was found that CPP may potentially decrease the amount of impurities in extracts of fucoidan, which would be beneficial for commercial production of fucoidan with a high level of purity. Moreover, the monosaccharide compositions of SH1–SH3 were measured and the results are presented in Table 2. Fucose, galactose, and mannose were shown to be the major monosaccharides in SH1–SH3. Smaller amounts of glucuronic acid, rhamnose, and glucose, as well as a tiny amount of xylose were also detected. Generally, CPP did not apparently affect the monosaccharide compositions of fucoidan for SH1–SH3. The fucoidan extracts obtained by SH1–SH3 were further characterized using Fourier transform infrared (FTIR) spectroscopy, nuclear magnetic resonance (NMR) spectroscopy, and molecular weight analyses. The FTIR results of SH1–SH3 shown in Figure 1A suggest that the typical signals for sulfated polysaccharides were obtained. The signals at 3401, 2941, 1230, and 1055 cm^−1^ correspond to the O–H stretching vibrations, the C–H stretching vibrations, the S=O asymmetric stretching vibration of the sulfate group, and the C–O–H in the glucosidal bond or C–O–C stretching vibrations in the ring [14,15]. The IR bands at 1621 and 1421 cm^−1^ arose from scissoring vibration of water and in-plane ring CCH, OCH and COH vibrations typical for polysaccharides. These bands may have contribution from antisymmetric and symmetric vibrations of carboxylate anions (COO^−^) in glucuronate units [14,15,16]. Bands at 900 and 837 cm^−1^ corresponded to C1–H bending vibration in β-anomeric units (probably galactose) and equatorial C2–O–S or C3–O–S bending vibrations in sulfate semi-esters. Other IR bands of sulfates are visible in Figure 1A near 620 and 580 cm^−1^. These bands were attributed to the anti-symmetric and symmetric O=S=O deformations [17]. Due to the similarity of the FTIR spectra in SH1–SH3, the structural aspects of sulfated polysaccharide and the positions of the sulfate group were not significantly altered by CPP. NMR spectroscopy is one of the most powerful techniques for analyzing the structure of complex polysaccharides [18]. In the present study, we utilized ^1^H-NMR spectra to evaluate whether the structural features among SH1–SH3 are different. The ^1^H-NMR spectra (Figure 1B) for SH1–SH3 revealed certain characteristic signals of fucoidan, which were in agreement with data reported by other investigators; for example, the anomeric signals at 5.5–5.0 ppm are consistent with the presence of l-fucopyranosyl units [19]. The signals at 4.57 and 4.46 ppm may be assigned to H-2 of a 2-sulfated fucopyranose residue [19]. The signal at 4.13 ppm (H-4) indicates 3-linked α-l-fucose [16]. The narrow signals at 3.9–3.6 ppm may be attributed to the presence of mannitol [20], a low-molecular metabolite of brown seaweeds [21] which may have been coextracted with our fucoidans. The detection of signals at 3.78 ppm (H-3) and 3.72 ppm (H-4) can be tentatively assigned to 4-linked β-d-galactose and 2,3-linked α-β-mannose, respectively [16]. The signals around 2.56 ppm may be tentatively assigned to the presence of glutamate [22,23], which may exist in the forms of amino acids or proteins coextracted with our fucoidans (Table 2). Several singlets around 2.2 ppm arose from methyl protons in *O*-acetyls [17,24]. These data are consistent with the findings reported by Bilan et al. (2004), which showed that algal polysaccharides are heterogeneous and branched, and certain additional constituents such as acetyl groups were also observed [24]. In addition, the signal at 1.32 ppm indicates the C6 methyl protons of L-fucopyranose [25]. In general, certain impurities such as mannitol and proteins are co-extracted with fucoidan extracts as revealed by NMR analyses. Most importantly, due to the similarity of the ^1^H-NMR spectra in SH1–SH3 (Figure 1B), it may be concluded that the structural features of fucoidan were not obviously altered by CPP. The high-performance liquid chromatography (HPLC) gel filtration analysis was utilized to characterize the molecular weights of SH1–SH3. The data presented in Figure 1C revealed that the average molecular weights of extracts for SH1 were 617.04 and 523.19 kDa; for SH2, the weights were 608.80 and 518.95 kDa; and for SH3, the weights were 601.20 and 384.50 kDa, respectively. The molecular weights of SH1–SH3 were reduced by raising the puffing pressures. These results suggest that CPP might have effects on the breakdown of the fucoidan backbone if high temperature and high pressure treatments are used as SH2 and SH3 appeared to result in a decrease of molecular weights. Taken together, CPP had certain effects including increased total sugar content, increased molar ratios of sulfate/fucose, decreased impurities of fucoidan, and decreased molecular weight. SH1–SH3 showed characteristics of fucoidan as elucidated by compositional, FTIR spectroscopy, NMR spectroscopy, and molecular weight analyses. In addition, SH3 contained the highest total sugar content and molar ratio of sulfate/fucose and the smallest molecular weight among SH1–SH3, and thus the biological functions of SH3 warrant further examination.

### 2.3. Antioxidant Activities for SH1, SH2, and SH3

Previous studies suggested that neuronal damage may be caused by an increase of free radical damage due to oxidative stress [2]. Thus, the antioxidant activities for SH1–SH3 warranted investigation. Three methodologies termed 2,2-diphenyl-1-picrylhydrazyl (DPPH), 2,2′-azino-bis(3-ethylbenzothiazoline-6-sulphonic acid) diammonium salt (ABTS), and ferric reducing antioxidant power (FRAP) analyses were applied in the present study to evaluate the antioxidant activities of SH1–SH3. Table 3 shows the IC_50_ of DPPH radical scavenging activity for SH1–SH3 ranged from 1.72 ± 0.07 to 2.58 ± 0.03 (mg/mL); the IC_50_ of ABTS^•+^ scavenging activity for SH1–SH3 ranged from 0.17 ± 0.01 to 0.34 ± 0.00 (mg/mL); and the FRAP values ranged from 13.10 ± 0.06 to 18.36 ± 0.11 (μmol vitamin C/g extract, dry basis). Since all fucoidan extracts SH1–SH3 exhibited antioxidant activities, further studies are needed to fully elucidate their neuroprotective functions.

### 2.4. Effects of SH1, SH2, and SH3 on 6-OHDA-Induced Apoptosis in SH-SY5Y Cells

**Figure 2:**
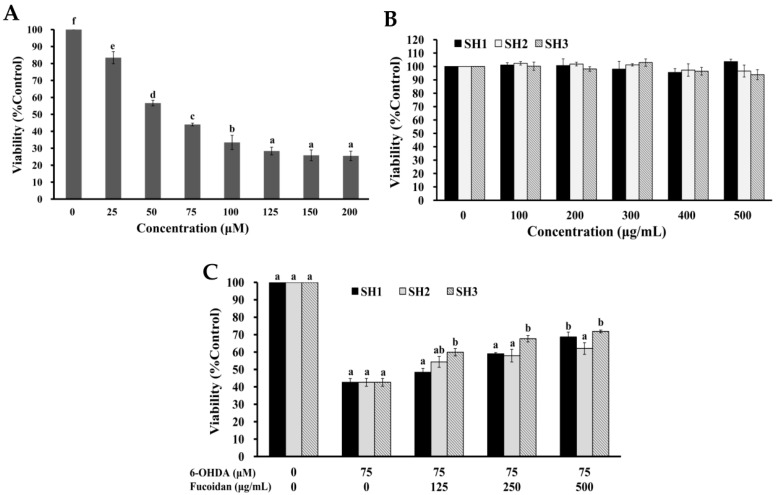
Effects of crude extracts of fucoidans (SH1, SH2, and SH3) and 6-OHDA treatment with or without SH1–SH3 pretreatment on cell viability of SH-SY5Y cells: (**A**) SH-SY5Y cells were treated with 6-OHDA (0–200 μM) for 24 h, and cell viability was assessed. Values are expressed as the mean ± SD (*n =* 3); (**B**) SH-SY5Y cells were treated with SH1, SH2, or SH3 (0–500 μg/mL) for 24 h, and cell viability was assessed. Values are expressed as the mean ± SD (*n =* 3); (**C**) SH-SY5Y cells were pretreated with SH1, SH2, or SH3 (0–500 μg/mL) for 6 h, followed by treatment with 75 μM 6-OHDA for 24 h, and cell viability was assessed. Values are expressed as the mean ± SD (*n =* 3). Different letters (in a, b, c, d, e, and f) mean statistically significant differences at *p* < 0.05. In each group of columns related to each concentration of fucoidan, the means that have at least one common letter do not differ significantly (*p* > 0.05).

**Figure 3:**
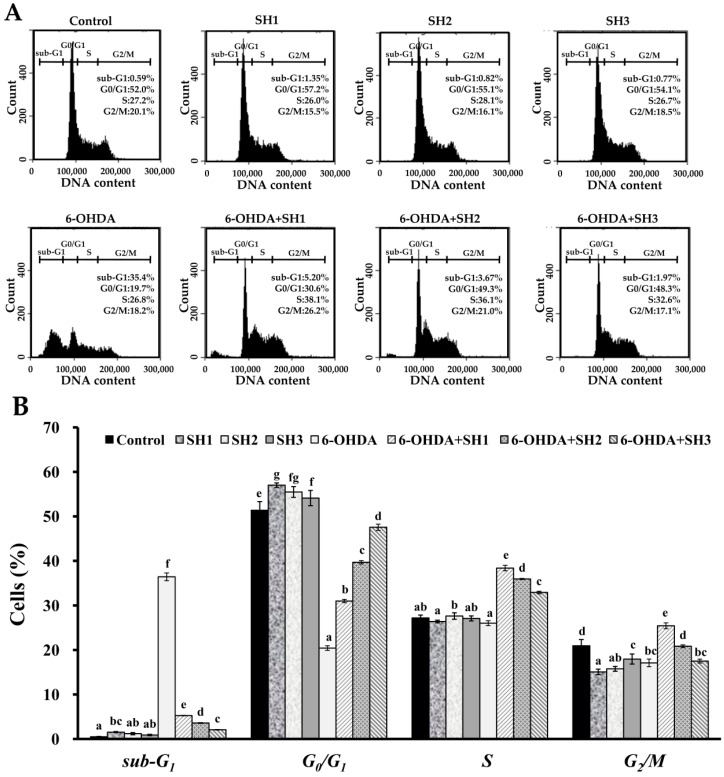
Effects of 6-OHDA treatment with or without SH1–SH3 pretreatment on cell cycle profiles of SH-SY5Y cells: (**A**) SH-SY5Y cells were pretreated with SH1, SH2, or SH3 at a concentration of 500 μg/mL for 6 h, followed by treatment with or without 75 μM 6-OHDA for 24 h, and cell cycle profiles were assessed; (**B**) summarizing bar graph of three cell cytometric analyses showing the percentages of cells in the *sub-G*_1_, *G*_0_*/G*_1_, *S*, and *G*_2_*/M* phases of the cell cycle according to treatments after analysis using BD Accuri C6 software. Results are shown as mean ± SD of three independent experiments. Different letters (in a, b, c, d, e, f, and g) mean statistically significant differences at *p* < 0.05. In each group of columns related to each cell cycle phase, differences exist between columns labeled with different letters, *p* < 0.05.

**Figure 4:**
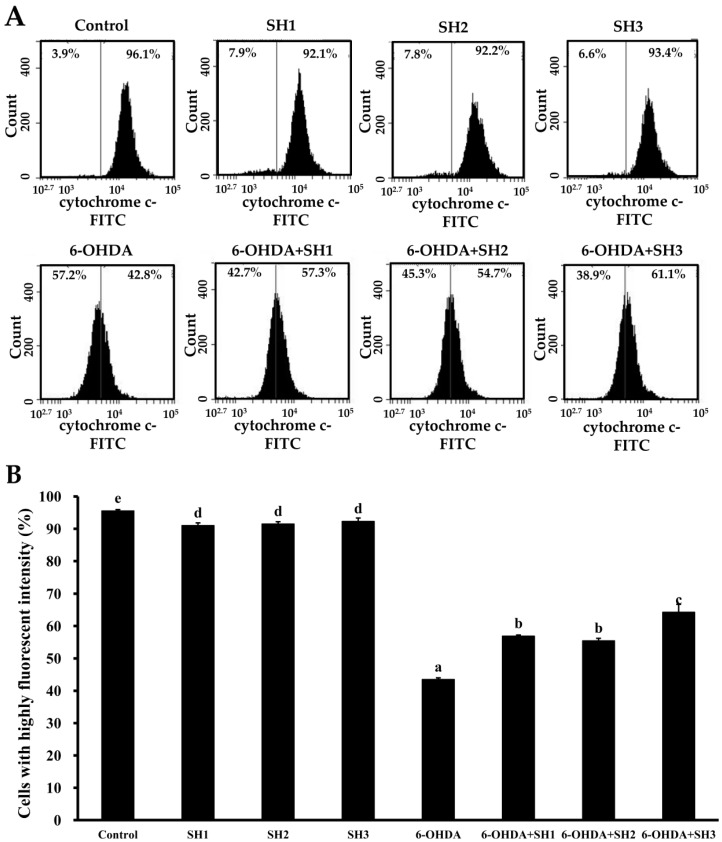
Effects of 6-OHDA treatment with or without SH1–SH3 pretreatment on the extent of cytochrome c release in SH-SY5Y cells: (**A**) SH-SY5Y cells were pretreated with SH1, SH2, or SH3 at a concentration of 500 μg/mL for 6 h, followed by treatment with or without 75 μM 6-OHDA for 24 h, and fluorescence histograms of immunolabeled cytochrome c were assessed; (**B**) summarizing bar graph of three cell cytometric analyses showing the percentages of cells with highly fluorescent intensity according to treatments after analysis using BD Accuri C6 software. Results are shown as mean ± SD of three independent experiments. Different letters (in a, b, c, d, and e) mean statistically significant differences at *p* < 0.05. Differences exist between columns labeled with different letters, *p* < 0.05.

**Figure 5:**
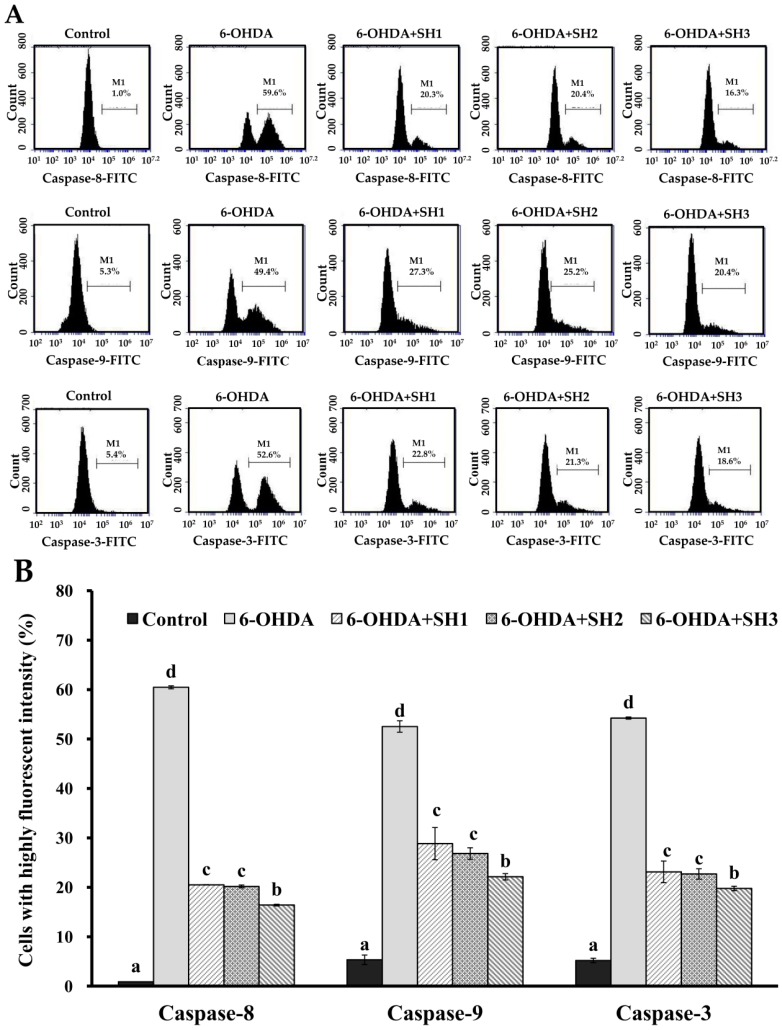
Effects of 6-OHDA treatment with or without SH1–SH3 pretreatment on active caspase-8, -9, and -3 in SH-SY5Y cells: (**A**) SH-SY5Y cells were pretreated with SH1, SH2, or SH3 at a concentration of 500 μg/mL for 6 h, followed by treatment with 75 μM 6-OHDA for 24 h, and fluorescence histograms of immunolabeled caspase-8, -9, and -3 were assessed; (**B**) summarizing bar graph of three cell cytometric analyses and showing the percentages of cells with highly fluorescent intensity according to treatments after analysis using BD Accuri C6 software. Results are shown as mean ± SD of three independent experiments. Different letters (in a, b, c, and d) mean statistically significant differences at *p* < 0.05. In each group of columns related to each type of caspase, differences exist between columns labeled with different letters, *p* < 0.05.

**Figure 6:**
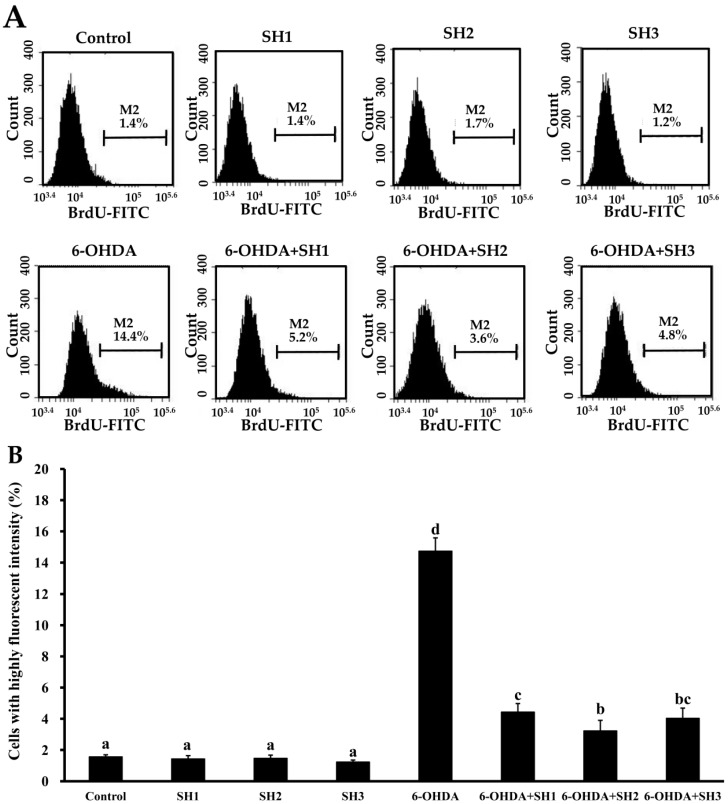
Effects of 6-OHDA treatment with or without SH1–SH3 pretreatment on the extent of DNA fragmentation in SH-SY5Y cells: (**A**) SH-SY5Y cells were pretreated with SH1, SH2, or SH3 at a concentration of 500 μg/mL for 6 h, followed by treatment with or without 75 μM 6-OHDA for 24 h, and fluorescence histograms of immunolabeled BrdU were assessed; (**B**) summarizing bar graph of three cell cytometric analyses showing the percentages of cells with highly fluorescent intensity according to treatments after analysis using BD Accuri C6 software. Results are shown as mean ± SD of three independent experiments. Different letters (in a, b, c, and d) mean statistically significant differences at *p* < 0.05. Differences exist between columns labeled with different letters, *p* < 0.05.

A variety of studies have provided evidence showing that neuronal apoptosis leads to the pathogenesis of acute and chronic neurodegenerative diseases in the adult brain following metabolic or neurotoxic insults [26,27]. PD is a well-known neurodegenerative disorder and is characterized by various symptoms including postural instability, gait abnormality, rest tremors, bradykinesia, and rigidity [28]. The potential mechanisms of PD-associated neurodegeneration can be explored by using an animal PD model. One of the neurotoxins which can induce PD in an animal model is 6-OHDA. 6-OHDA, an oxidative metabolite of dopamine, can either undergo extracellular autooxidation or intracellular enzymatic oxidation through the monoamine oxidase type B, yielding ROS and quinolinic products [29]. Thus, 6-OHDA has been broadly used to generate experimental models of PD [30]. An in vitro model widely used in PD research is the neuroblastoma SH-SY5Y cell line [31]. Both undifferentiated and differentiated SH-SY5Y cells have been utilized for in vitro experiments requiring neuronal-like cells. In general, the most common method employed for the differentiation of SH-SY5Y cells is the addition of retinoic acid (RA) in concentrations ranging from 5 μM to 100 μM, and for a period of time from 1 day up to 21 days [31]; generally, the process is complicated. Sometimes, the RA treatment may also influence the expression of neuronal and dopaminergic markers and affect the susceptibility to dopaminergic neurotoxins in SH-SY5Y cells [32,33]. In addition, it was reported that 784 out of the 962 papers used no differentiation protocols in SH-SY5Y experiments [31]. Moreover, Cheung et al. reported that RA-differentiation conferred higher tolerance in SH-SY5Y cells, potentially by up-regulating survival signaling, including the Akt pathway, and thus the actual toxicity induced by 6-OHDA cannot be revealed in RA-differentiated cells. They therefore suggested that undifferentiated SH-SY5Y is more appropriate for studying neurotoxicity or neuroprotection in experimental Parkinson’s disease research [33]. As such, undifferentiated SH-SY5Y cells were adopted in the present study. Here, we examined the protective effects of SH1–SH3 on 6-OHDA-induced dopaminergic neuronal cell death in SH-SY5Y cells. When SH-SY5Y cells were treated with various concentrations of 6-OHDA, the cell viability was decreased in response to the incremental increases in 6-OHDA concentration (Figure 2A). The treatment of SH-SY5Y cells with 75 μM 6-OHDA for 24 h decreased the cell viability and the value was 44.01% ± 0.83% of the control group (Figure 2A). Thus, the concentration of 75 μM 6-OHDA was utilized for further cellular experiments. To evaluate the cytotoxic effects of SH1–SH3 on SH-SY5Y cells, the cells were treated with different concentrations of SH1–SH3 for 24 h, and then the cell viability of SH-SY5Y cells was evaluated. None of the fucoidan extracts SH1–SH3 exhibited cytotoxicity to SH-SY5Y cells at concentrations from 0 to 500 μg/mL (Figure 2B). In addition, the treatment of SH-SY5Y cells with 75 μM 6-OHDA for 24 h decreased the cell viability, and pretreatment of SH-SY5Y cells with SH1–SH3 at concentrations of 125–500 μg/mL for 6 h dose-dependently attenuated 6-OHDA-induced cellular cytotoxicity (Figure 2C). In general, SH3 had a greater effect on reversion of 6-OHDA-induced cytotoxicity as compared to SH1 and SH2. 6-OHDA is known as an apoptosis inducer in various cell types [34]. In order to further examine the protective effects of SH1–SH3 on 6-OHDA-induced apoptosis in SH-SY5Y cells, the cell cycle distribution, cytochrome c release, activation of caspase-8, -9, and -3, and DNA fragmentation were performed by flow cytometry. The flow cytometry is capable of detecting apoptotic dead cells and cells with fragmented nuclei (also called *sub-G*_1_ cells) when stained with propidium iodide (PI) [35]. The results in Figure 3A,B show that, in the cell populations of the *sub-G*_1_, *G*_0_*/G*_1_, *S*, and *G*_2_*/M* phases, the fucoidan extracts SH1–SH3 had a similar cell cycle distribution profile to that of SH-SY5Y cells as compared to the control. When SH-SY5Y cells were treated with 75 μM 6-OHDA, a significant increase (36.45% ± 0.88%, *p* < 0.05) in the proportion of cells with *sub-G*_1_ DNA content as compared to control cells (0.49% ± 0.07%). Additionally, decreased cell populations of *G*_0_*/G*_1_, *S*, and *G*_2_*/M* phases in the 6-OHDA-treated cells were also observed as compared to control cells (Figure 3A,B). These results suggest that 6-OHDA caused SH-SY5Y cell cycle arrest at the *sub-G*_1_ phase. Moreover, treatment of SH-SY5Y cells with 75 μM 6-OHDA in the presence of 500 μg/mL SH1, SH2, or SH3 significantly attenuated the *sub-G*_1_ populations to 5.25% ± 0.05%, 3.57% ± 0.10%, and 2.07% ± 0.07%, respectively (*p* < 0.05). These findings suggest that 6-OHDA could obviously increase the percentage of cells with *sub-G*_1_ DNA content, an indicator of greater DNA fragmentation as well as apoptosis or cell cycle arrest at the *sub-G*_1_ phase. In addition, treatment of cells with SH1, SH2, or SH3 significantly attenuated the *sub-G*_1_ populations of 6-OHDA-induced apoptotic cells, indicating that SH1, SH2, and SH3 could protect SH-SY5Y cells against neurotoxicity. We also found that SH3 was the most effective at reducing the *sub-G*_1_ population in 6-OHDA-treated SH-SY5Y cells, followed by SH2, and then SH1. Cytochrome c release from mitochondria to cytosol is a hallmark of apoptosis and is often utilized to characterize the mitochondria-dependent apoptotic pathway [36]. Here, an immunodetection of cytochrome c by flow cytometry was applied to quantify release of cytochrome c in cells. As shown in Figure 4A,B, the fucoidan extracts SH1–SH3 possessed a similar cytometric profile to that of SH-SY5Y cells as compared to the control. When cells were treated with 75 μM 6-OHDA, the population of high fluorescent cells decreased from 96.80% ± 0.22% (control) to 43.47% ± 0.53%, indicating cytochrome c underwent release from mitochondria. In contrast, when cells were treated with 75 μM 6-OHDA in the presence of 500 μg/mL SH1, SH2, or SH3, the populations of high fluorescent cells were significantly increased to 56.90% ± 0.33%, 55.43% ± 0.77%, and 64.27% ± 2.46%, respectively. These results clearly indicate that SH1–SH3 may protect SH-SY5Y cells from the 6-OHDA-induced mitochondria-dependent apoptotic effect. In addition, SH3 was more effective at preventing 6-OHDA-induced cytochrome c release in cells than SH1 and SH2. There are two basic mechanisms involved in the induction of apoptosis, the extrinsic pathway (death receptor-mediated) and the intrinsic pathway (regulated at the level of the mitochondria). The extrinsic pathway of apoptosis signals through cell surface molecules like Fas/FasL resulting in the recruitment of the Fas-associated death domain (FADD). The FADD associates with Fas and recruits pro-caspase-8. This active complex is referred to as the death-inducing signaling complex (DISC). The DISC activates caspase-3, and then apoptosis occurs. The intrinsic pathway occurs following loss of mitochondrial transmembrane potential (MTP), resulting in the release of cytochrome c into the cytoplasm. Cytochrome c forms a complex with the cytoplasmic protein Apaf-1 and pro-caspase-9 that is termed the “apoptosome” that results in activation of caspase-3, leading to systematic disassembly of the cell [37]. Thus, the activation of caspases such as caspase-8, -9, and -3 is the typical hallmark of apoptosis. Here, we measured active caspase-8, -9, and -3 in cells using a flow cytometric-based analysis. As shown in Figure 5A,B, when cells were treated with 75 μM 6-OHDA, the population of high fluorescent cells for the caspase-8 group increased from 0.87% ± 0.09% (control) to 60.47% ± 0.78%, indicating the augmentation of active caspase-8. Conversely, when cells were treated with 75 μM 6-OHDA in the presence of 500 μg/mL SH1, SH2, or SH3, the population of high fluorescent cells was significantly decreased to 20.50% ± 0.22%, 20.17% ± 0.33%, and 16.40% ± 0.37%, respectively. A similar tendency was also observed in the caspase-9 and caspase-3 groups. These results clearly indicate that SH1–SH3 may protect SH-SY5Y cells from 6-OHDA-induced activation of caspase-8, -9, and -3. In addition, SH3 was more effective at preventing 6-OHDA-induced activation of caspase-8, -9, and -3 in cells than SH1 and SH2. In the event of apoptosis, both the extrinsic pathway and the intrinsic pathway result in the activation of caspase-3. Caspase-3 seems to play a central role in apoptotic events and is specifically required for DNA fragmentation leading to the typical apoptotic pattern of DNA laddering [38,39]. Therefore, the detection of DNA fragmentation (a hallmark of late-stage apoptosis) in cells was performed by flow cytometric-based terminal deoxynucleotidyl transferase-mediated dUTP nick end-labeling (TUNEL) assay. As shown in Figure 6A,B, the fucoidan extracts SH1–SH3 possessed a similar cytometric profile to that of SH-SY5Y cells as compared to the control. When cells were treated with 75 μM 6-OHDA, the population of high fluorescent cells significantly increased from 1.57% ± 0.12% (control) to 14.73% ± 0.85%, indicating the occurrence of DNA fragmentation. In contrast, when cells were treated with 75 μM 6-OHDA in the presence of 500 μg/mL SH1, SH2, or SH3, the population of high fluorescent cells was significantly decreased to 4.43% ± 0.54%, 3.23% ± 0.66%, and 4.03% ± 0.65%, respectively. These results clearly show that SH1–SH3 may protect SH-SY5Y cells from 6-OHDA-induced DNA fragmentation of cells. In summary, all of the treated fucoidan extracts SH1–SH3 protected SH-SY5Y cells from 6-OHDA-induced apoptosis as illustrated by the cell cycle distribution, cytochrome c release, activation of caspase-8, -9, and -3, and DNA fragmentation analyses. Among SH1–SH3, SH3 generally exhibited the greatest neuroprotective effects. The neuroprotective effects of SH3 may be positively correlated to the high total sugar content, high molar ratio of sulfate/fucose, and low molecular weight in SH3. Further experimental studies using differentiated SH-SY5Y cells or in vivo models are needed to elucidate the precise mechanisms involved. Since SH3 had a relatively high extraction yield, a relatively low level of impurities, and the greatest neuroprotective effects, we thus suggest that SH3 could be a good candidate for application in the preventive therapy of neurodegenerative diseases, especially PD.

### 2.5. Phosphorylation of Akt Is Involved in the Neuroprotective Effects of SH1, SH2, and SH3 on 6-OHDA-Induced Apoptosis in SH-SY5Y Cells

**Figure 7:**
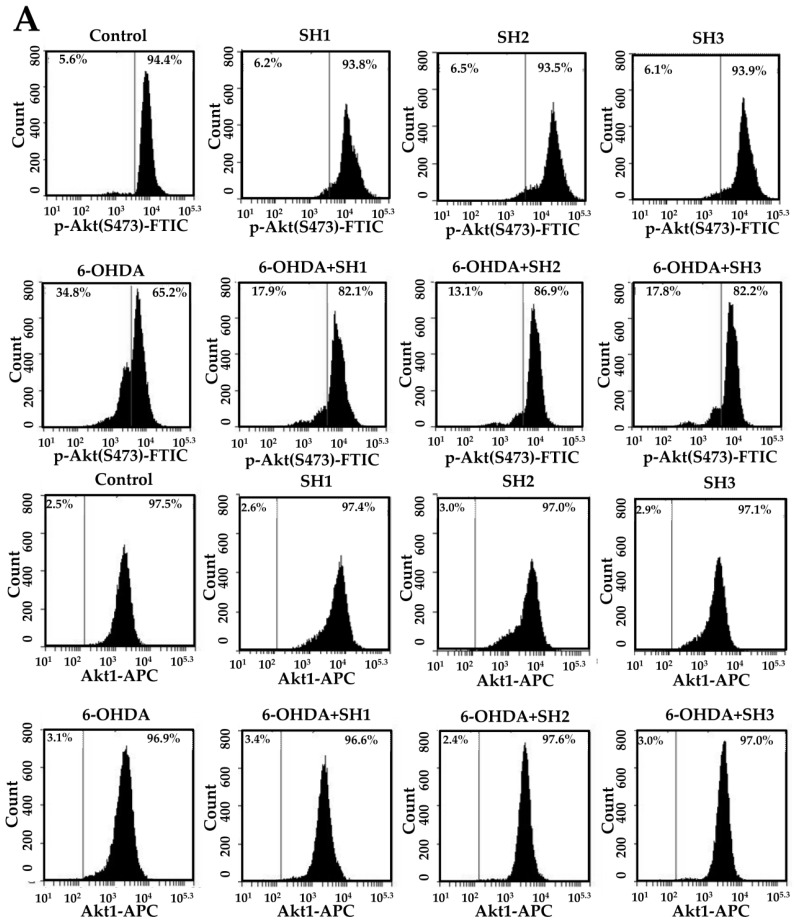

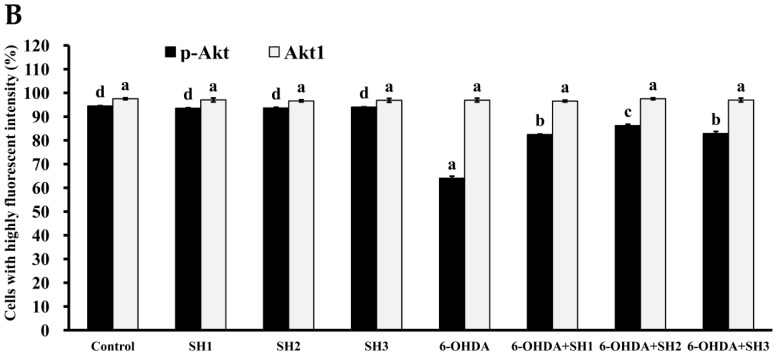
Effects of 6-OHDA treatment with or without SH1–SH3 pretreatment on the expression of p-Akt and Akt1 in SH-SY5Y cells: (**A**) SH-SY5Y cells were pretreated with SH1, SH2, or SH3 at a concentration of 500 μg/mL for 6 h, followed by treatment with or without 75 μM 6-OHDA for 24 h, and fluorescence histograms of immunolabeled p-Akt and Akt1 were assessed; (**B**) summarizing bar graph of three cell cytometric analyses showing the percentages of cells with highly fluorescent intensity according to treatments after analysis using BD Accuri C6 software. Results are shown as mean ± SD of three independent experiments. Different letters (in a, b, c, and d) mean statistically significant differences at *p* < 0.05. In each group of columns related to p-Akt or Akt1, differences exist between columns labeled with different letters, *p* < 0.05.

PI3Ks/protein kinase B (PKB or Akt) pathway has vital roles in the differentiation, survival, cell cycle, metabolism, cytokine production, growth, and activation of B cells [40]. Previous studies suggested that the PI3K/Akt pathway is involved in the prevention of apoptotic cell death in a PD model of SH-SY5Y cells [41]. We thus evaluated the expressions of phosphorylated Akt (p-Akt) and total Akt (Akt1) in cells by flow cytometry. As shown in Figure 7A,B, it was found that fucoidan extracts SH1–SH3 possessed a cytometric profile of p-Akt and Akt1 expressions that was similar to that of SH-SY5Y cells as compared to the control. When cells were treated with 75 μM 6-OHDA, the population of high fluorescent cells significantly decreased from 94.50% ± 0.14% (control) to 64.10% ± 0.79%, indicating the occurrence of dephosphorylation of Akt. Conversely, when cells were treated with 75 μM 6-OHDA in the presence of 500 μg/mL SH1, SH2, or SH3, the population of high fluorescent cells was significantly increased to 82.43% ± 0.24%, 86.27% ± 0.53%, and 82.90% ± 0.85%, respectively. These results clearly indicate that SH1–SH3 may protect SH-SY5Y cells from 6-OHDA-induced dephosphorylation of Akt in cells. In addition, when examining the expression of Akt1, it was found the expression level of Akt1 was not changed with respect to different treatments. Our results provide evidence that phosphorylation of Akt is involved in the preventive effects of SH1–SH3 on 6-OHDA-induced neurotoxicity. Further elucidation of the molecular mechanism and signaling cascade underlying the neuroprotective effects of SH1–SH3 (especially SH3) is warranted.

## 3. Materials and Methods

### 3.1. Materials and Chemicals

A sample of *S. hemiphyllum* (SH) was collected from Pingtung, Taiwan and then washed with fresh water, oven-dried at 50 °C, and kept in plastic bags at 4 °C until further experiments. l-fucose, l-rhamnose, d-glucose, d-glucuronic acid, d-galactose, d-mannose, d-xylose, d-galacturonic acid, gallic acid, sodium carbonate, potassium sulfate, DPPH, ABTS, 2,4,6-tris(2-pyridyl)-*s*-triazine (TPTZ), sodium acetate trihydrate, thioglycolic acid solution, 3-(4,5-dimethylthiazol-2-yl)-2,5-diphenyltetrazolium bromide (MTT), 6-OHDA, bovine serum albumin (BSA), Bradford reagent, and dimethyl sulfoxide (DMSO) were obtained from Sigma-Aldrich (St. Louis, MO, USA). 2,2,2-Trifluoroacetic acid (TFA) was obtained from Panreac (Barcelona, Spain). Potassium persulfate, potassium bromide (KBr), and sodium sulfite were purchased from Merck (Darmstadt, Germany). Fetal bovine serum (FBS), trypsin/EDTA, penicillin, and streptomycin were obtained from Gibco Laboratories (Grand Island, NY, USA). Dulbecco’s Modification of Eagle’s Medium/Ham’s F-12 50/50 Mix medium was obtained from Corning (Corning, NY, USA). All other reagents were of analytical grade or the best grade available.

### 3.2. Compressional-Puffing Procedure

The dried algal sample (weight = 2.7 g, H_2_O = 12.9%) was puffed according to our previously described procedure [6,7]. The detailed operational parameters are described in Table 1.

### 3.3. Water Extraction Procedure

The water extraction procedure for obtaining fucoidan was performed according to our previously described protocol [7] with slight modification. In brief, the compressional-puffed algal sample was mixed with 95% ethanol (*w*/*v* = 1:10), shaken for 4 h at room temperature to remove pigments, proteins and lipid, and then centrifuged at 970× *g* for 10 min. The residue was collected, mixed with double-distilled water (*w*/*v* = 1:10) and placed in a water bath kept at 85 °C for 1 h with shaking (120 rpm) to extract the polysaccharides. The mixture was centrifuged at 3870× *g* for 10 min and the supernatant was collected. 2% (*w*/*v*) CaCl_2_ solution was added to the supernatant for 1 h with shaking (120 rpm) to precipitate alginate. The mixture was centrifuged at 3870× *g* for 10 min, and the supernatant was ultrafiltrated (300× *g* for 10 min) using 100 kDa membrane for protein and calcium removal. The retentate was collected and ethanol (95%) was added into the supernatant to give a final ethanol concentration of 20% in order to further precipitate alginic acid. The mixture was centrifugated at 9170× *g* for 30 min, the supernatant was collected, and 95% ethanol was added until a final ethanol concentration of 50% was reached in order to obtain fucoidan precipitate. The ethanol-precipitated fucoidan was then recovered by centrifugation at 9170× *g* for 30 min and lyophilized. Extraction yield was calculated using the following equation:

Extraction yield (%) = (*g*_A_/*g*_B_) × 100 (1)

where *g*_A_ represents the weight of the extracted solid on a dry basis, and *g*_B_ is the weight of the sample on a dry basis.

### 3.4. Chemical Methods

The determinations of crude protein, fat, moisture, and ash were carried out using the following AOAC procedures. Carbohydrate was calculated as weight differences between the total weight and the sum of the amounts of moisture, protein, lipid, and ash. The phenol-sulfuric acid colorimetric method was used to determine the total sugar content, and using l-fucose as the standard. The fucose content was determined by the method of Gibbons [42], and l-fucose was used as the standard. Protein in the extract was quantified by the Bradford method using BSA as the standard. Uronic acids were estimated by the colorimetric method using d-galacturonic acid as the standard [43]. Alginate content was measured according to the method of Honya [44]. Polyphenols were analyzed by the Folin-Ciocalteu method and gallic acid was used as the standard. Sulfate content of polysaccharide was determined by first hydrolyzing the polysaccharide with 1 N HCl solution for 5 h at 105 °C. The hydrolysate was then quantified based on the percentage of sulfate composition using Dionex ICS-1500 Ion Chromatography (Sunnyvale, CA, USA) with an IonPac AS9-HC column (4 × 250 mm) at a flow rate of 1 mL/min at 30 °C with conductometric detection. The eluent was 9 mM Na_2_CO_3_, and K_2_SO_4_ was utilized as the standard.

### 3.5. Analysis of Monosaccharide Composition

The monosaccharide composition of polysaccharide was analyzed according to our previously described method [7], using l-fucose, d-xylose, d-galactose, d-glucose, d-glucuronic acid, l-rhamnose, and d-mannose as the standards.

### 3.6. NMR Spectroscopy

A 20 mg polysaccharide sample was dissolved in 550 μL of 99.9% deuterium oxide (D_2_O) in a NMR tube and ^1^H NMR spectrum was recorded at 60 °C on a Varian 400-MR (400 MHz) spectrometer (Agilent Technologies, Santa Clara, CA, USA) for proton detection. The proton chemical shift was expressed in parts per million (ppm).

### 3.7. Molecular Weight Analysis

The molecular weight analysis of the polysaccharides was conducted according to the method of Yang [7]. The standards used to calibrate the column were various dextrans with different molecular weights (50, 150, and 670 kDa), which were obtained from Sigma-Aldrich (Sigma-Aldrich, St. Louis, MO, USA).

### 3.8. FTIR Spectroscopy

The FTIR analysis was performed according to the method of Huang [45]. In brief, polysaccharide and KBr (*w*/*w*, 1:50) were mixed and ground evenly until particles measured less than 2.5 μm in size. The transparent KBr pellets were made at 500 kg/cm^2^ under vacuum. The FTIR spectra were obtained using a FT-730 spectrometer (Horiba, Kyoto, Japan), and the absorbance was read between 400 and 4000 cm^−1^. Pellet of KBr alone was used as a background.

### 3.9. DPPH Radical Scavenging Activity

The DPPH radical scavenging activity was determined according to a method described elsewhere [35]. Briefly, 50 µL of sample was added to 150 µL 0.1 mM freshly prepared DPPH solution (in methanol). The mixture was shaken vigorously for 1 min, left to stand for 30 min in the dark at room temperature, and the absorbance of all sample solutions was measured at 517 nm using an ELISA reader (PowerWave 340, Bio-Tek Instruments, Winooski, VT, USA). The radical-scavenging activity was calculated using the following Equation:

(2) $$\mathrm{DPPHradical}-\mathrm{scavenging}\;\left(\%\right)=\left[1-\frac{A_{\mathrm{sample}}}{A_{\mathrm{control}}}\;\right]\;\times \;100$$

where A_sample_ is the absorbance of the methanol solution of DPPH with tested samples, and A_control_ represents the absorbance of the methanol solution of DPPH without the sample.

### 3.10. ABTS Radical Cation Scavenging Activity

The scavenging activity of the samples against ABTS radical cation was measured according to a method described elsewhere [35]. In brief, the ABTS^•+^ solution was prepared by reacting 5 mL of ABTS solution (7 mM) with of 88 µL of potassium persulfate (140 mM), and the mixture was kept in the dark at room temperature for 16 h. The solution was diluted with 95% ethanol to obtain an absorbance of 0.70 ± 0.05 at 734 nm. To start the assay, 100 µL diluted ABTS^•+^ solution was mixed with 100 µL of various sample solutions. The mixture was allowed to react at room temperature for 6 min, and the absorbance of all sample solutions at 734 nm was measured using an ELISA reader (PowerWave 340, Bio-Tek Instruments, Winooski, VT, USA). The blank was prepared in the same manner, except that distilled water was used instead of the sample. The activity of scavenging ABTS^•+^ was calculated according to the following Equation:

(3) $$\mathrm{ABTScation}\;\mathrm{radical}-\mathrm{scavenging}\;\left(\%\right)=\left[1-\frac{A_{\mathrm{sample}}}{A_{\mathrm{control}}}\;\right]\times \;100$$

where A_sample_ is the absorbance of ABTS with tested samples, and A_control_ represents the absorbance of ABTS without the sample.

### 3.11. FRAP Assay

The FRAP assay was performed according to the protocol proposed by Benzie and Strain [46]. Briefly, the FRAP solution was prepared by adjusting 10 mL of acetate buffer (300 mM) to pH 3.6 via the addition of acetic acid. It was then mixed with 1 mL of ferric chloride hexahydrate (20 mM) dissolved in distilled water and 1 mL of TPTZ (10 mM) dissolved in HCl (40 mM). A volume measuring 25 μL of the test sample dissolved at different concentrations was prepared. Freshly prepared FRAP solution (175 μL) warmed at 37 °C was added to the sample, while the same volume of acetate buffer was utilized as the blank. The absorbance at 593 nm was monitored by an ELISA reader (PowerWave 340, Bio-Tek Instruments, Winooski, VT, USA). Vitamin C was used as a standard and FRAP values were expressed as micromole vitamin C equivalents per gram of sample on a dry basis (μmol vitamin C/g sample, dry basis).

### 3.12. Cell Culture

A SH-SY5Y cell line (human dopaminergic, neuroblastoma, ATCC^®^ CRL-2266™) was obtained from the Food Industry Research and Development Institute, Hsinchu, Taiwan. SH-SY5Y cells were cultured in Dulbecco’s Modification of Eagle’s Medium/Ham’s F-12 50/50 Mix medium supplemented with 10% FBS, plus streptomycin (100 μg/mL), and penicillin (100 units/mL) at 37 °C in the presence of 95% air, and 5% CO_2_. The culture medium was changed every 48–72 h.

### 3.13. Cell Viability Test

Cell viability was measured by quantitative colorimetric MTT assay [35]. Briefly, SH-SY5Y cells (1 × 10^5^/mL in a 96-well plate) were plated with culture medium and incubated for 24 h at 37 °C, with 5% CO_2_ in a humidified atmosphere. The cells were then incubated with test compounds at various concentrations for various times. The reaction was stopped by removing the treatment media, adding MTT reagent (0.1 mg/mL), and then allowing the reagent to react at 37 °C in 5% CO_2_ for 2 h. MTT was removed, and cells were lysed with DMSO. The absorbance at 570 nm was measured using an ELISA reader (PowerWave 340, Bio-Tek Instruments, Winooski, VT, USA). The cell viability (%) was calculated using the following Equation:

(4) $$\mathrm{Cell}\;\mathrm{viability}\;\left(\%\right)=\left(\frac{T}{C}\right)\times \;100$$

where T is the absorbance in the test, and C is the absorbance for the control.

### 3.14. Cell Cycle Analysis

Cells were seeded in a 6-cm dish at 4 × 10^4^ cells/mL in 5 mL growth medium and incubated for 24 h at 37 °C, with 5% CO_2_ in a humidified atmosphere. Afterwards, cells were pretreated with or without different fucoidans at a final concentration of 500 μg/mL for 6 h, and then treated with or without 6-OHDA at a final concentration of 75 μM for 24 h. After treatment, the cells (including floating and adherent cells) were harvested and washed with 1× ice-cold phosphate-buffered saline (PBS) twice. The cell pellets were collected and fixed in 70% ice-cold ethanol solution and stored in the freezer for at least 2 h. After washing with staining buffer twice, cells were then resuspended in a solution containing 50 μg/mL PI and 25 μg/mL RNase A at 37 °C for 15 min. The stained cells were then transferred to flow tubes and the DNA contents in the cells were evaluated by a BD Accuri C6 flow cytometer (BD Biosciences, San Jose, CA, USA) and a minimum of 10,000 cells per sample was collected. The percentage of each phase in the cell cycle was determined by BD Accuri C6 software.

### 3.15. Cytochrome c Release Analysis

Cells were seeded in 6-cm dish at 4 × 10^4^ cells/mL in 5 mL growth medium and incubated for 24 h at 37 °C, with 5% CO_2_ in a humidified atmosphere. Afterwards, cells were pretreated with or without different fucoidans at a final concentration of 500 μg/mL for 6 h, and then treated with or without 6-OHDA at a final concentration of 75 μM for 24 h. After treatment, the cells (including floating and adherent cells) were harvested and washed with 1× ice-cold PBS twice. The cell pellets were collected, resuspended with 100 μL 1× PBS, and 100 μL fixation buffer was then added, followed by storage at 37 °C for 20–60 min in the dark. Next the cells were washed with 2 mL 1× permeabilization buffer twice, and the cells were resuspended with 100 μL 1× permeabilization buffer, followed by addition of 10 μL FITC (fluorescein isothiocyanate)-labelled anti-cytochrome c antibody (Thermo Fisher Scientific, Waltham, MA, USA), and incubation at room temperature for 60 min in the dark. After washing with 2 mL 1× permeabilization buffer, the cells were centrifugated, and resuspended in 2 mL staining buffer. The stained cells were then transferred to flow tubes and analyzed by a BD Accuri C6 flow cytometer (BD Biosciences, San Jose, CA, USA) and a minimum of 10,000 cells per sample were collected. The results were calculated by BD Accuri C6 software.

### 3.16. Activated Caspase-8, -9, and -3 Analyses

The activation state of caspase-8, -9 and -3 was evaluated by using the CaspGLOW^TM^ fluorescein active caspase staining kit (Thermo Fisher Scientific, Waltham, MA, USA). Briefly, cells were seeded in a 6-cm dish at 4 × 10^4^ cells/mL in 5 mL growth medium and incubated for 24 h at 37 °C, with 5% CO_2_ in a humidified atmosphere. Afterwards, cells were pretreated with or without different fucoidans at a final concentration of 500 μg/mL for 6 h, and then exposed to 6-OHDA at a final concentration of 75 μM for 24 h. After treatment, the cells (including floating and adherent cells) were harvested and washed with 1× ice-cold PBS twice. The cell pellets were collected and the cell density was adjusted to 1 × 10^6^ cells/mL with complete medium and then incubated with 1 μL FITC-IETD-FMK (for caspase-8 detection), FITC-LEHD-FMK (for caspase-9 detection), or FITC-DEVD-FMK (for caspase-3 detection) at 37 °C, with 5% CO_2_ for 60 min in the dark. After washing with 0.5 mL wash buffer twice, the stained cells were transferred to flow tubes and analyzed by a BD Accuri C6 flow cytometer (BD Biosciences, San Jose, CA, USA) and a minimum of 10,000 cells per sample were collected. The data were calculated by BD Accuri C6 software.

### 3.17. Quantitation of DNA Fragmentation by TUNEL Assay

DNA fragmentation was evaluated using the Apo-BrdU apoptosis detection kit (Thermo Fisher Scientific, Waltham, MA, USA). Briefly, cells were seeded in a 6-cm dish at 4 × 10^4^ cells/mL in 5 mL growth medium and incubated for 24 h at 37 °C, with 5% CO_2_ in a humidified atmosphere. Afterwards, cells were pretreated with or without different fucoidans at a final concentration of 500 μg/mL for 6 h, and then treated with or without 6-OHDA at a final concentration of 75 μM for 24 h. After treatment, the cells (including floating and adherent cells) were harvested and washed with 1× ice-cold PBS twice. The cell pellets were collected and fixed with 4% paraformaldehyde followed by 70% ice-cold ethanol and then stored in the freezer for at least 12 h. After washing with 1 mL wash buffer twice, the fragmented DNA in apoptotic cells were labeled with BrdU followed by incubation with FITC-conjugated anti-BrdU antibody at room temperature for 30 min in the dark. The stained cells were then transferred to flow tubes and analyzed by a BD Accuri C6 flow cytometer (BD Biosciences, San Jose, CA, USA) and a minimum of 10,000 cells per sample were collected. The data were calculated by BD Accuri C6 software.

### 3.18. Phosphorylated Akt Analysis 

Cells were seeded in a 6-cm dish at 4 × 10^4^ cells/mL in 5 mL growth medium and incubated for 24 h at 37 °C, with 5% CO_2_ in a humidified atmosphere. Afterwards, cells were pretreated with or without different fucoidans at a final concentration of 500 μg/mL for 6 h, and then treated with or without 6-OHDA at a final concentration of 75 μM for 24 h. After treatment, the cells (including floating and adherent cells) were harvested and washed with 1× ice-cold PBS twice. The cell pellets were collected, resuspended with 100 μL 1× PBS, and 100 μL fixation buffer was then added, followed by storage at 37 °C for 20–60 min in the dark. Then 1 mL 90–100% ice-cold methanol was added and the cells were incubated at 2–8 °C for at least 30 min. Next, the cells were washed with 5 mL staining buffer twice, resuspended with 100 μL staining buffer, and 2 μL APC (allophycocyanin)-conjugated anti-Akt1 antibody (Thermo Fisher Scientific, Waltham, MA, USA) and 5 μL FITC-conjugated anti-phospho-Akt (Ser473) antibody (Thermo Fisher Scientific, Waltham, MA, USA) were added, followed by incubation at room temperature for 60 min in the dark. After washing with 2 mL 1× staining buffer twice, the stained cells were transferred to flow tubes and analyzed by a BD Accuri C6 flow cytometer (BD Biosciences, San Jose, CA, USA) and a minimum of 10,000 cells per sample were collected. The results were determined by BD Accuri C6 software.

### 3.19. Statistical Analysis

Results are presented as means ± standard deviation (SD) of three independent experiments. Data were analyzed using the Statistical Package for the Social Sciences (SPSS). The results obtained were analyzed using one-way analysis of variance (ANOVA), followed by Duncan’s Multiple Range tests. Data were considered statistically different at *p* < 0.05.

## 4. Conclusions

In this paper, we extracted fucoidans from SH using CPP followed by hot water extraction. CPP increased the extraction yield, total sugar content, and molar ratios of sulfate/fucose of fucoidan, and decreased the molecular weight and impurities of fucoidan. All extracts (SH1–SH3) showed characteristics of fucoidan by compositional, FTIR spectroscopy, NMR spectroscopy, and molecular weight analyses. SH1–SH3 showed antioxidant activities by DPPH, ABTS, and FRAP analyses. Fucoidan extracts SH1–SH3 protected SH-SY5Y cells from 6-OHDA-induced apoptosis as illustrated by the cell cycle distribution, cytochrome c release, activation of caspase-8, -9, and -3, and DNA fragmentation analyses. Among SH1–SH3, SH3 exhibited a relatively high extraction yield, the lowest levels of impurities, and was the most effective at reversing the neurotoxicity of SH-SY5Y cells induced by 6-OHDA. We therefore suggest that SH3 may be a good candidate for future development as a therapeutic agent for preventive therapy of neurodegenerative diseases, especially PD. We also demonstrated that phosphorylation of Akt is involved in the preventive effects of SH1–SH3 on 6-OHDA-induced neurotoxicity. Further elucidation of the molecular mechanism and the signaling cascade involved in the neuroprotective effects of SH1–SH3 (especially SH3) using differentiated SH-SY5Y cells or in vivo models is warranted.

## Figures and Tables

**Table 1:** Parameters for compressional-puffing, degree of moisture loss of puffed algal samples, and extraction yields of fucoidan for SH1, SH2, and SH3.

Variables of Compressional-Puffing		SH1	SH2	SH3
Mechanical compression	Pressure (kg/cm^2^)	0	5	5
	Number of compression times	0	3	3
Puffing	Temperature (°C)	0	140	180
	Pressure (kg/cm^2^)	0	1.7	10.0
Reaction time	Time (s)	0	10	10
Degree of moisture loss of puffed algal samples		SH1 ^1^	SH2 ^1^	SH3 ^1^
Degree of moisture loss (%)		0.00 ± 0.00 ^a^	16.21 ± 1.17 ^b^	29.56 ± 2.21 ^c^
Variables of water extraction		SH1	SH2	SH3
Extraction temperature	Temperature (°C)	85	85	85
Extraction time	Time (h)	1	1	1
Extraction yields of fucoidan		SH1 ^1^	SH2 ^1^	SH3 ^1^
Extraction yield (%)		1.51 ± 0.09 ^a^	1.93 ± 0.28 ^ab^	2.06 ± 0.14 ^b^

^1^ Values are mean ± SD (*n* = 3); means in the same row followed by the different letters (in ^a^, ^b^, and ^c^) are significantly different (*p* < 0.05).

**Table 2:** Compositional analyses for SH1, SH2, and SH3.

**Chemical Composition**	**SH1** **^2^**	**SH2** **^2^**	**SH3** **^2^**
Total sugar (%)	53.67 ± 2.59 ^a^	58.83 ± 1.61 ^b^	68.83 ± 1.91 ^c^
Uronic acid (%)	5.66 ± 0.08 ^a^	7.04 ± 0.22 ^c^	6.12 ± 0.08 ^b^
Fucose (%)	24.53 ± 1.15 ^a^	22.76 ± 0.38 ^a^	23.87 ± 1.76 ^a^
Sulfate (%)	18.87 ± 1.65 ^b^	15.58 ± 0.16 ^a^	24.16 ± 2.80 ^c^
Sulfate/fucose (molar ratio) ^1^	1.32 ± 0.11 ^a^	1.17 ± 0.01 ^a^	1.74 ± 0.20 ^b^
Protein (%)	2.28 ± 0.05 ^c^	2.05 ± 0.12 ^b^	1.83 ± 0.07 ^a^
Polyphenols (%)	1.25 ± 0.08 ^c^	1.06 ± 0.06 ^b^	0.71 ± 0.06 ^a^
Monosaccharide compositions (molar ratio)	SH1	SH2	SH3
Fucose	1.00	1.00	1.00
Mannose	0.32	0.27	0.28
Rhamnose	0.15	0.15	0.14
Glucose	0.09	0.07	0.06
Glucuronic acid	0.21	0.20	0.20
Galactose	0.40	0.35	0.35
Xylose	ND ^3^	0.02	0.02

^1^ Sulfate/fucose (molar ratio) = (weight of sulfate/molecular weight of sulfate)/(weight of fucose/molecular weight of fucose); ^2^ values are mean ± SD (*n =* 3); means in the same row followed by the different letters (in ^a^, ^b^, and ^c^) are significantly different (*p* < 0.05); ^3^ ND: not detected.

**Table 3:** Antioxidant activities for SH1, SH2, and SH3.

Antioxidant Activity	SH1 ^2^	SH2 ^2^	SH3 ^2^
DPPH/IC_50_ (mg/mL) ^1^	1.72 ± 0.07 ^a^	2.42 ± 0.15 ^b^	2.58 ± 0.03 ^b^
ABTS/IC_50_ (mg/mL) ^1^	0.17 ± 0.01 ^a^	0.26 ± 0.00 ^b^	0.34 ± 0.00 ^c^
FRAP/vitamin C equivalent (μmol vitamin C/g extract, dry basis)	18.36 ± 0.11 ^c^	15.81 ± 0.24 ^b^	13.10 ± 0.06 ^a^

^1^ IC_50_ value: concentration of fucoidan capable of scavenging 50% of DPPH or ABTS free radicals; ^2^ values are mean ± SD (*n* = 3); means in the same row followed by the different letters (in ^a^, ^b^, and ^c^) are significantly different (*p* < 0.05).
